# Hypomethylation induced overexpression of PLOD3 facilitates colorectal cancer progression through TM9SF4-mediated autophagy

**DOI:** 10.1038/s41419-025-07503-5

**Published:** 2025-03-25

**Authors:** Renzhong Zhu, Chuanxin Tian, Nan Gao, Zhiqiang Li, Sheng Yang, Yue Zhang, Ming Zhou, Kangpeng Jin, Chuan Zhang, Yueming Sun

**Affiliations:** 1 Institute of Translational Medicine, Medical College, Yangzhou University, Yangzhou, China; 2 Department of General Surgery, The First Affiliated Hospital of Nanjing Medical University, Nanjing, China; 3 The Colorectal Institute of Nanjing Medical University, Nanjing, China; 4 Jiangsu Province Engineering Research Center of Colorectal Cancer Precision Medicine and Translational Medicine, Nanjing, China; 5 General Surgery department of Dongtai People’s Hospital, Yancheng, China; 6 MOE Key Laboratory of Bioinformatics, Center for Synthetic and Systematic Biology, School of Life Sciences, Tsinghua University, Beijing, China

**Keywords:** Colorectal cancer

## Abstract

Colorectal cancer (CRC) ranks among the primary causes of human mortality globally. Numerous studies have highlighted the significant role of PLOD3 in the progression of various cancers. However, the exact function and underlying mechanisms of PLOD3 in CRC remains incompletely understood. To investigate the expression of PLOD3, qRT‒PCR, immunohistochemistry and western blotting were utilized to analyze the expression of PLOD3 in CRC tissues and adjacent normal tissues. Functional assays were conducted to assess the roles of PLOD3 both in vitro and in vivo. To elucidate the potential mechanism of PLOD3 in CRC, a range of techniques, including coimmunoprecipitation, immunofluorescence, CHX pulse-chase, and ubiquitination assays were used. As the results indicated, hypomethylation of the PLOD3 promoter leads to its over- expression in CRC, and elevated PLOD3 levels are associated with a poor prognosis. Both in vitro and in vivo models demonstrated that PLOD3 enhances CRC cell proliferation, invasion, and migration. Furthermore, through mechanistic studies, TM9SF4 was identified as a protein that interacts with PLOD3 and contributes to CRC progression by promoting autophagy. Additionally, PLOD3 could be secreted by CRC cells and secreted PLOD3 could promote CRC cells migration and invasion. These results demonstrated that PLOD3 promotes CRC progression through the PLOD3/TM9SF4 axis and could be a potential biomarker and treatment target for CRC.

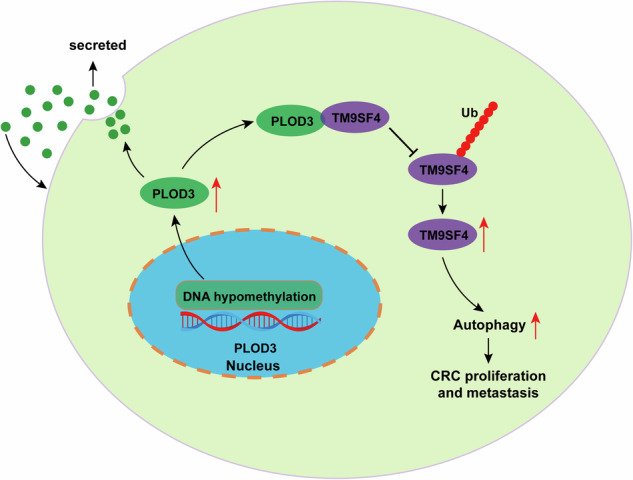

## Introduction

Colorectal cancer (CRC) is the most common gastrointestinal carcinoma and has high morbidity and mortality rates globally [1], ranking fourth and second, respectively, among all cancers in China [2]. By 2030, the burden of CRC is expected to increase by 60% worldwide, which represents a severe challenge to human health [3]. Due to the lack of effective detection indicators for early diagnosis, approximately 25% of CRC patients are diagnosed at an advanced stage with metastasis [4]. Although numerous methods have been implemented for treating CRC in clinical settings, including surgery, chemo-radiotherapy, and immunotherapy, however, the outcomes for advanced CRC patients, particularly those with distant metastases, remain unsatisfactory. The five-year survival rate for these patients is dishearteningly low, falling below 5% [5]. Consequently, identifying novel biomarkers for early detection and further exploring the mechanism underlying CRC progression are urgently needed to provide a theoretical and experimental basis for the diagnosis and treatment of CRC.

The extracellular matrix (ECM) is a highly dynamic structure involved in all tissues that functions in maintaining tissue integrity and modulating diverse functions of cells, such as proliferation and migration [6]. Multiple studies have demonstrated that dysregulation of the ECM contributes to a series of pathological conditions, including invasive cancer [7–9]. Collagen, an abundant and crucial component of the ECM [10], undergoes aberrant deposition and cross-linking, leading to ECM remodeling and increased stiffness. This, in turn, transforms the phenotype of the cells [11]. Therefore, dysregulation of the enzyme that catalyzes collagen synthesis could theoretically dampen or promote cancer progression. Collagen biosynthesis and maturation are mainly dependent on lysine residue hydroxylation, a process catalyzed by the procollagen-lysine, 2-oxoglutarate 5-dioxygenase family (PLODs), which is a crucial biochemical reaction. The PLOD family comprises three members: PLOD1, PLOD2, and PLOD3 [12]. Currently, numerous studies have established that the expression of PLOD1 and PLOD2 is elevated in CRC and that these genes could serve as potential biomarkers [13, 14], suggesting that PLOD3, the only isozyme responsible for collagen synthesis and maturation [15], might also play a significant role in the progression of CRC. Despite reports indicating increased expression of PLOD3 in various cancers, for instance, suppressing PLOD3 expression has been found to hinder lung cancer progression by modulating the PKC-delta signaling pathway [16]. Similarly, it has been shown to enhance the sensitivity of HER-2-positive gastric cancer to trastuzumab treatment via the FoxO3/Survivin pathway [17]. Elevated PLOD3 expression has also been detected in liver cancer and is associated with a poor prognosis [18]. However, the specific role of PLOD3 in CRC has yet to be fully elucidated. Given its potential significance in cancer progression, further investigation into the function of PLOD3 in CRC is warranted. Such studies may provide valuable insights into the pathophysiology of CRC and pave the way for novel therapeutic strategies targeting PLOD3.

Autophagy is a highly conserved process that occurs in eukaryotes and involves the degradation and recycling of intracellular substances. It plays a fundamental role in cellular homeostasis [19]. Numerous studies have demonstrated that dysregulation of autophagy is definitively correlated with multiple diseases [20–22]. Cancer, characterized by a greater energy demand during its initiation and development, theoretically has a closer relationship with autophagy. Although a growing number of studies have suggested that autophagy can either promote or inhibit cancer progression [23, 24], the etiological links between cancer and autophagy remain controversial. Previous studies have indicated that PLOD3 may exert its biological function partially through the autophagy pathway; however, the exact mechanism involved remains to be elucidated.

In this study, we investigated the role of PLOD3 in CRC. We found that PLOD3 is overexpressed in CRC due to promoter hypomethylation, and its increased expression is positively correlated with tumor stage, lymph node metastasis, and distant metastasis. Functionally, PLOD3 enhances CRC cell proliferation, migration, and invasion Mechanistically, PLOD3 prevents the ubiquitination and degradation of TM9SF4, thereby stabilizing its protein level and enhancing autophagy, which further contributes to the progression of CRC. Our findings provide novel insights into therapeutic strategies for CRC.

## Materials and methods

### Cell lines and culture

The human CRC cell lines SW480, RKO, HCT116, and DLD-1; the normal human colon mucosal epithelial cell line NCM460; and the human embryonic kidney cell line HEK293T were purchased from the Cell Bank of Type Culture Collection of the Chinese Academy of Sciences (Shanghai, China). All cell lines were cultured in RPMI-1640 medium supplemented with 10% fetal bovine serum (FBS; Gibco), and 1% penicillin/streptomycin in a moist atmosphere containing 5% CO_2_ at 37 °C.

### Data collection and processing

All samples were obtained from CRC patients at the First Affiliated Hospital of Nanjing Medical University from 2017 to 2018. Tissues were immediately stored in 4% formalin after surgery. The tissue microarray was generated by Servicebio (Wuhan, China) containing formalin-fixed, paraffin-embedded tissues of 80 CRC patients. None of the patients received neoadjuvant chemoradiotherapy before surgery, and the study was approved by the Institutional Ethical Board of our hospital (2022-SRFA-142). Bulk RNA-sequencing datasets and corresponding clinical information were obtained from The Cancer Genome Atlas (TCGA) Colon Adenocarcinoma (COAD; *n* = 452) and two Gene Expression Omnibus (GEO) datasets: GSE29621 (*n* = 65) and GSE17536 (*n* = 177).

### Gene set enrichment analysis

Gene Set Enrichment Analysis (GSEA) was conducted to identify enriched pathways with PLOD3 related genes. PLOD3-related genes were identified by performing Pearson correlation analysis, selecting the top 1000 genes most strongly correlated with PLOD3. Only genes with a statistically significant correlation (*p* < 0.05) were included in the analysis. The GSEA analysis was based on KEGG pathway annotations, and significantly enriched pathways were selected based on their adjusted p-values. The bar plot was generated to display the count of genes associated with each pathway, color-coded by their adjusted p-values. The clusterProfile package (v4.2.2) was utilized to analyze and compare enriched GO Biological Process (GO BP) pathways between groups with PLOD3 high and PLOD3 low expression levels of specific genes. To visualize the pathways of interest, the gseaNb function from the GseaVis package (v0.0.1) was applied.

### RNA extraction and quantitative real-time PCR

Total RNA was extracted using TRIzol reagent (Invitrogen, Carlsbad, CA) according to the manufacturer’s instructions. Total RNA was reverse transcribed to cDNA using HiScript RT Mix (Vazyme, Jiangsu, China). A SYBR Premix Ex Taq Kit (TaKaRa Biotechnology, Dalian, China) was used to perform qRT‒PCR. The data were analyzed by StepOne software v2.3. The primers used for qRT‒PCR are shown in Table S1.

### Western blot analysis and antibodies

Proteins were extracted from cells according to the manufactory’smanufacturer’s protocol (KeyGEN BioTech). The protein concentration in the cell lysates was quantified using the BCA Protein Assay Kit (Beyotime Biotechnology). WB was performed as previously reported [25]. The primary antibodies used are listed in Table S2.

### Cell transfection

The lentivirus-delivered shRNAs targeting PLOD3 and TM9SF4 were synthesized by Genomedi-

tech (Shanghai, China). The full-length sequences of PLOD3 and TM9SF4 with a 3xFlag tag synthesized by Genomeditech were subcloned and inserted into the lentiviral vector (2488-PGMLV-SB3:PGMLV-hU6-MCS-CMV-Puro-WPRE). Ubiquitin overexpression plasmids were obtained from Obio (Shanghai, China). The shRNAs and plasmids were transfected with Lipofectamine 3000 (Invitrogen) as a vector. The transfection efficiency was confirmed by qRT‒PCR and western blotting. The sequences of the shRNAs are shown in Table S1.

### Cell proliferation assays

Cell Counting Kit-8 (CCK-8) (Beyotime, Shanghai, China) and colony formation assays were performed to detect cell proliferation as described previously [26]. Proliferation was analyzed using the mean number of cells in three fields for each sample.

### Transwell and scratch wound healing assays

Transwell and scratch wound healing assays were conducted as reported previously [27]. Three random fields were selected and measured using microscopy.

### Flow cytometry assay of apoptosis

A total of 3 × 10^5^ cells were seeded on 6-well plates and cultured for 72 h. The cells were then washed twice with PBS and resuspended. Then, Annexin V-APC and 7-AAD staining were performed using an Annexin V APC/7-AAD Apoptosis Detection Kit (MultiSciences, Shanghai, China) according to the manufacturer’s instructions. Eventually, FlowJo (BD Biosciences, USA) was used to analyze the percentage of apoptotic CRC cells.

### Immunohistochemistry (IHC)

IHC staining was conducted by Servicebio (Wuhan, China) using a TMA to assess the protein levels of PLOD3 (histochemistry score = ∑(pi × *i*)). Then, protein expression was divided into high expression and low expression according to the medium H-score. The details of the antibodies used in this study are listed in Table S2.

### Transmission electron microscopy (TEM)

Transmission electron microscopy was used to measure the number of autophagosomes in CRC cells. The treated CRC cells were collected and fixed with 2.5% glutaraldehyde at 4 °C overnight. The cells were washed with PBS three times and postfixed with 1% osmium tetroxide for 2 h. Graded ethanol was used to dehydrate the sections, which were then infiltrated with propylene oxide. Finally, after the slides were stained with uranyl acetate and lead citrate, TEM (Hitachi, Ltd., Tokyo, Japan) was applied to visualize the samples.

### Coimmunoprecipitation (Co-IP)

The physical binding of PLOD3 to TM9SF4 was investigated using an IP/Co-IP Kit (#88804, Thermo Fisher Scientific). Cells transfected with the indicated plasmids were lysed, and the cell lysates were mixed with primary antibody homogeneously and incubated at 4 °C for 12 h. Then, the immunocomplex and flag beads were incubated for 1.5 h at room temperature. After that, the cells were washed twice with IP lysis buffer, and the immunocomplexes were subsequently washed once with RNase-free water. The samples were boiled for 10 min after elution with 1× SDS loading buffer. Western blotting was performed to evaluate the immunoprecipitated proteins. The primary antibodies used are listed in Table S2.

### Immunofluorescence (IF)

The detailed procedure of the immunofluorescence staining assay has been previously described [28]. The antibodies utilized in this study are listed in Table S3. To assess autophagy flux, cells expressing RFP-GFP-LC3B were treated with DAPI for 15 min following fixation. Subsequently, a confocal microscope was used to directly observe the cells.

### Ubiquitination assay

Stably transfected cells were incubated with the proteasome inhibitor MG132 (Beyotime, Shanghai, China) at a concentration of 20 μM for 8 h prior to collection. The cell lysates were then incubated with a specific antibody for coimmunoprecipitation to measure the level of ubiquitination.

### Methylation-specific PCR (MSP) and bisulfite sequencing PCR (BSP) analysis

Both MSP and BSP were used to evaluate the PLOD3 DNA methylation level in CRC and adjacent normal tissues. Genomic DNA was extracted using the DNeasy Blood & Tissue Kit (Qiagen) in accordance with the manufacturer’s guidelines. Following extraction, the collected genomic DNA (2 μg) was treated with bisulfite DNA lysis buffer for 1 h at 37 °C. The bisulfite-modified DNA was then utilized for further analysis. Primer sequences for MSP and BSP are shown in Table S1.

### Enzyme-linked immunosorbent assay (ELISA)

The concentration of PLOD3 in cell-free conditioned media from CRC cells, serum from CRC patients and healthy individuals were assayed with a human PLOD3 ELISA Kit (Shanghai ZCbio Technology Co., Ltd.) according to the manufacturer’s instructions.

### AOM/DSS murine model

An AOM/DSS murine model was established using 6-week-old male C57BL/6 mice divided into three per groups. On the first day, AOM (Sigma, USA) was intraperitoneally injected at a concentration of 10 mg/kg. Seven days later, the mice were fed 2.5% DSS (MP Biomedicals, USA) dissolved in drinking water for one week, followed by 2 weeks of regular drinking water. This cycle was repeated twice, and then the mice were given regular drinking water until day 112, when they were sacrificed.

### Xenografts

All animals used in this study were approved by the Nanjing Medical University Ethics Committee (IACUC-2407078). Six-week-old male nude mice (BALB/c) were utilized for the subcutaneous tumor formation and metastasis models. With respect to the xenograft model, 1×10^6^ stably transfected DLD-1 and SW480 cells, along with their parallel control cells, were subcutaneously injected into the bilateral groin of the mice. The tumor volumes were measured every 5 days. Twenty-five days after the injection, the mice were sacrificed, and the tumors were dissected. To detect the metastasis capability of CRC cells, 100 µl suspensions containing 1 × 10^6^ luciferase-labeled transfected CRC cells were injected into the distal tip of the spleen or the tail veins of mice to construct liver or lung metastasis models. Five weeks later, images of the metastatic foci were obtained with an IVIS 100 Imaging System (Xenogen, USA) after D-luciferin (150 mg/kg) (Goldbio, USA) was intraperitoneally injected into the mice. Then, the mice were sacrificed under anesthesia, and the livers and lungs were dissected for further study.

### Statistical analysis

Each assay was repeated more than three times independently. GraphPad Prism 9.0 (La Jolla, CA, USA) and SPSS software (version 23.0, IBM Corp, Armonk, NY, USA) were used to perform the statistical analysis. Independent Student’s t test, ANOVA, and the chi-square test were applied to determine statistical significance in this study. K‒M curves were used to show OS. The data are shown as the means ± standard deviations (SD). *P* < 0.05 was considered to indicate statistical significance (*P < 0.05, **P < 0.01, ***P < 0.001, ****P < 0.0001).

## Results

### PLOD3 is significantly overexpressed in CRC tissue and indicates poor prognosis

**Fig. 1:**
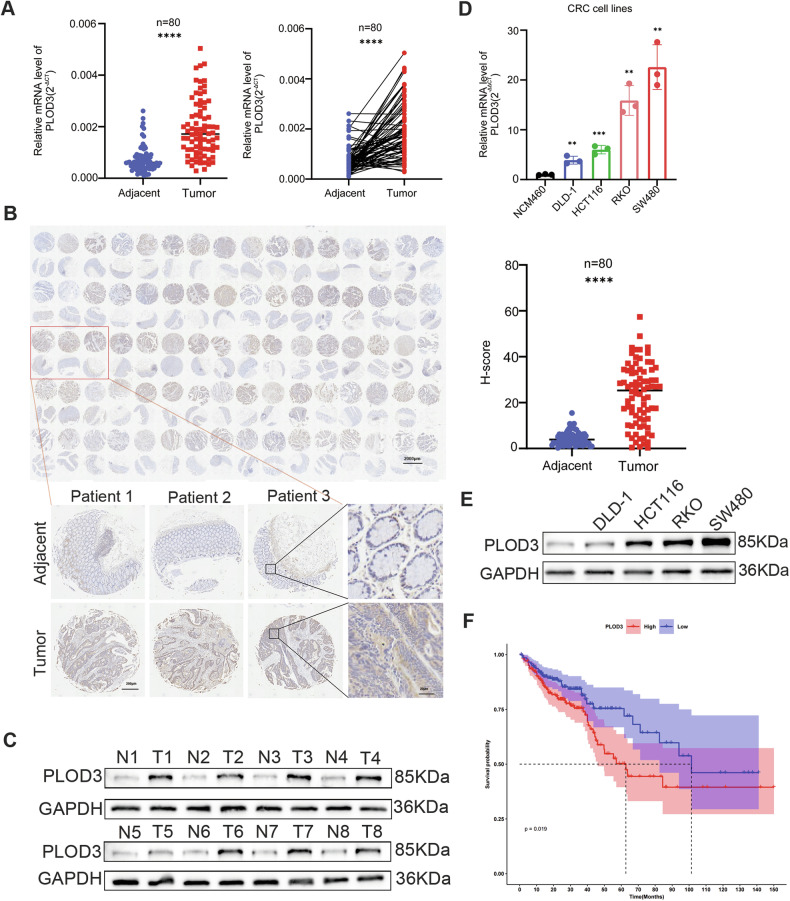
Both the mRNA and Protein level of PLOD3 were upregulated in CRC. **A** The mRNA level of PLOD3 was evaluated by qRT‒PCR in 80 CRC tissues and matched adjacent tissues. **B** The protein level of PLOD3 was measured by IHC in the TMA containing 80 CRC patients’ samples. **C** The protein level of PLOD3 were detected by WB in 8 fresh matched CRC tumor and adjacent tissues. **D**, **E** The mRNA and protein levels of PLOD3 were determined in CRC cell lines and NCM460 cells by qRT‒PCR and WB. F. PLOD3 overexpressed indicated a poor prognosis of CRC patient by K-Mcurve survival analysis. Data are presented as the mean ± SD at least three independent experiments, *P < 0.05, **P < 0.01, ***P < 0.001, ****P < 0.0001 and *P* > 0.05, not significant (n.s.).

To explore the role of PLOD3 in CRC, qRT‒PCR was performed to evaluate the PLOD3 mRNA levels in 80 CRC tissues and paired normal tissues. PLOD3 was overexpressed in CRC tissues compared with paired normal tissues (Fig. 1A), which was consistent with the findings in the TCGA and GEO datasets (Fig. S1A). Next, immunohistochemistry (IHC) showed that PLOD3 was upregulated in CRC tissues, as reflected by the increased H-scores obtained (Fig. 1B), which is consistent with the Clinical Proteomic Tumor Analysis Consortium (CPTAC) data (Fig. S1B). In addition, we performed western blotting to measure the protein levels in 8 fresh CRC tissue samples and their paracancerous tissues. As expected, the protein levels in CRC tissues were significantly greater than those in their paracancerous tissues (Fig. 1C). In addition, both the mRNA and protein levels of PLOD3 were obviously upregulated in CRC cell lines compared with those in a normal human colon mucosal epithelial cell line (NCM460) (Fig. 1D, E). Furthermore, we investigated the correlation between the expression of PLOD3 and clinicopathological characteristics of CRC patients. Interestingly, the expression profile of PLOD3 was found to be positively correlated with nervous invasion, tumor stage, lymph node metastasis, and distant metastasis in CRC patients (Table 1). This association becomes particularly evident when analyzing specific clinical subgroups, N1 + N2 group with lymph node metastasis, M1 group with distant metastasis, and III + IV tumor stage group all exhibited significantly greater levels of PLOD3 expression (Fig. S1D–F). These findings implicate PLOD3 in facilitating the progression of CRC. Furthermore, Kaplan‒Meier survival analysis demonstrated that elevated expression of PLOD3 was associated with a poorer prognosis for CRC patients (Fig. 1F). Collectively, these results strongly suggest that PLOD3 is upregulated in CRC and that its high expression is linked to unfavorable outcomes. These findings indicate that PLOD3 may serve as a potential biomarker for early CRC detection and prognosis prediction.

**Table 1:** Association between the expression of PLOD3 and clinicopathology of CRC patients.

	all patients	PLOD3		*P* value
		High	Low	
All case	80	43 (53.75%)	37 (46.25%)	
Age(years)				
60	23	12 (*15%*)	11 (13.75%)	
≥60	57	31 (38.75%)	26 (32.5%)	0.857
Genders				
Male	50	26 (32.5%)	24 (30%)	
Female	30	17 (*21.25%*)	13 (16.25%)	0.685
Tumor site				
Colon	35	16 (20%)	19 (23.75%)	
Rectal	45	27 (33.75%)	18 (22.5%)	0.204
Tumor size				
5 cm	49	23 (28.75%)	26 (32.5%)	
≥5 cm	31	20 (25%)	11 (13.75%)	0.124
Tumor Stage				
I + II	43	16 (20%)	27 (33.8%)	
III + IV	37	27 (33.7%)	10 (12.5%)	0.001*
Lymph node metastasis				
Yes	34	26 (32.5%)	8 (10%)	
No	46	17 (21.25%)	29 (36.25%)	<0.001*
Vascular invasion				
Yes	19	12 (15%)	7 (8.75%)	
No	61	31 (38.75%)	30 (37.5%)	0.346
Nervous invasion				
Yes	14	11 (13.75%)	3 (3.75%)	
No	66	32 (40%)	34 (42.5%)	0.04*
Distant metastasis				
Yes	17	14 (17.5%)	3 (3.75%)	
No	63	29 (36.25%)	34 (42.5%)	0.008*
CEA (ng/ml)				
5	49	24 (*30%*)	25 (31.25%)	
≥5	31	19 (23.75%)	12 (15%)	0.282

The bold type represents *P* values < 0.05.

*P* < 0.05 was considered significant.

### PLOD3 is upregulated by promoter CpG hypomethylation in CRC

**Fig. 2:**
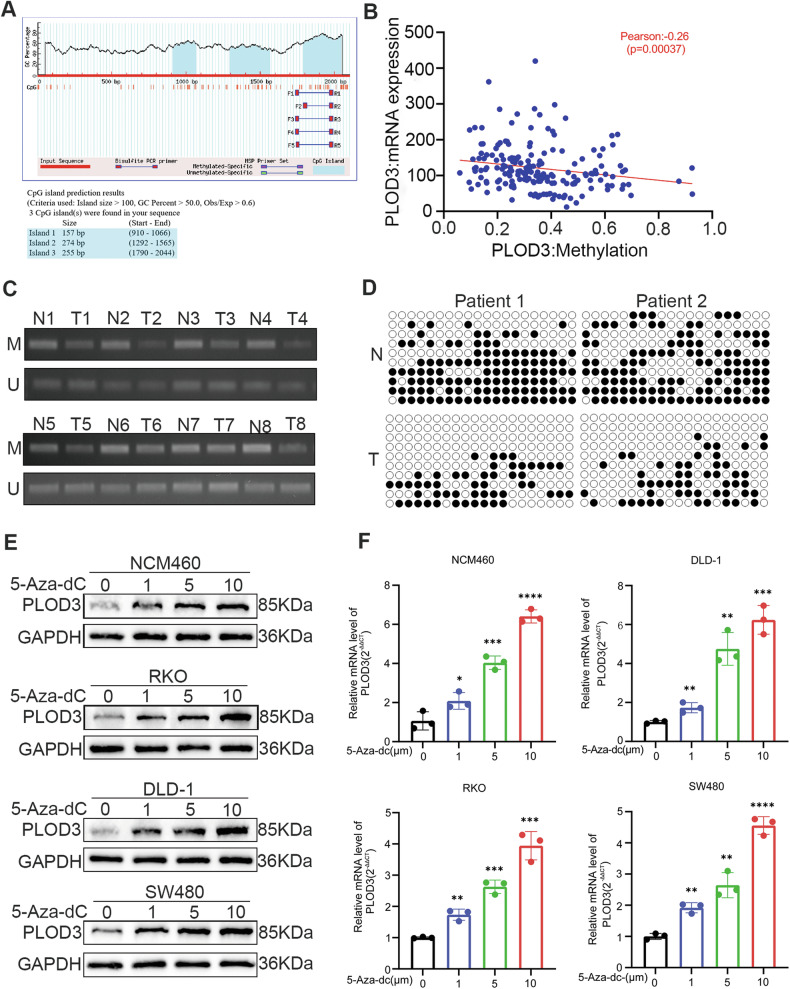
DNA methylation contributes to PLOD3 over expression. **A** CpG islands were predicted in the PLOD3 promoter sequence. **B** TCGA database was employed to evaluate the correlation between PLOD3 mRNA expression and DNA methylation levels. **C** methylation-specific PCR(MSP) was performed to detect the PLOD3 methylation levels in eight randomly selected pairs of CRC tissues and corresponding adjacent tissues. M, methylated; U, unmethylated. **D** bisulfite sequencing PCR(BSP) was conducted to assessed the DNA methylation status of 8 randomly selected pairs of CRC tissues and corresponding adjacent tissues. **E** Increasing expression of PLOD3 protein levels in CRC cells incubated with different concentration of 5-Aza-dC. **F** Increasing expression of PLOD3 mRNA levels in CRC cells incubated with different concentration of 5-Aza-dC. All data are shown as the mean ± SD of three independent biological replicates. *P < 0.05, ****P < 0.0001 and *P* > 0.05, not significant (n.s.).

Previous studies have established that hypomethylation of DNA promoter can contribute to oncogene upregulation [29]. Consistent with this finding, we initially predicted CpG islands within the PLOD3 promoter sequence by utilizing the MethPrimer program, as the results indicated that it contains 3 typical CpG islands (Fig. 2A). Subsequently, we investigated the relationship between the PLOD3 mRNA level and CpG hypomethylation to elucidate the mechanism underlying the upregulation of PLOD3 in CRC, island 3 was selected and the data derived from the TCGA database were analysised. As the result shows, a significant negative correlation between PLOD3 mRNA expression and DNA methylation was detected (Pearson correlation coefficient = **−**0.26; *P* = 0.00037) (Fig. 2B). These findings suggest that the overexpression of PLOD3 might be modulated by promoter hypomethylation. Consequently, to further investigate the methylation status of the PLOD3 promoter in CRC specimens, we employed methylation-specific PCR (MSP) and bisulfite sequencing PCR (BSP) assays. The results revealed that the PLOD3 promoter in CRC tissues was hypomethylated compared to that in paired adjacent tissues (Fig. 2C, D). Moreover, after the cells were treated with 5-Aza, a DNA methyltransferase inhibitor, the expression of PLOD3 was restored in NCM460 cells, and PLOD3 expression increased in a concentration-dependent manner in CRC cell lines at both the mRNA and protein levels (Fig. 2E, F). In summary, our findings suggest a potential link between PLOD3 expression and DNA methylation levels in CRC, suggesting a possible epigenetic regulatory mechanism.

### PLOD3 promotes the proliferation, migration, and invasion of CRC cells in vitro

**Fig. 3:**
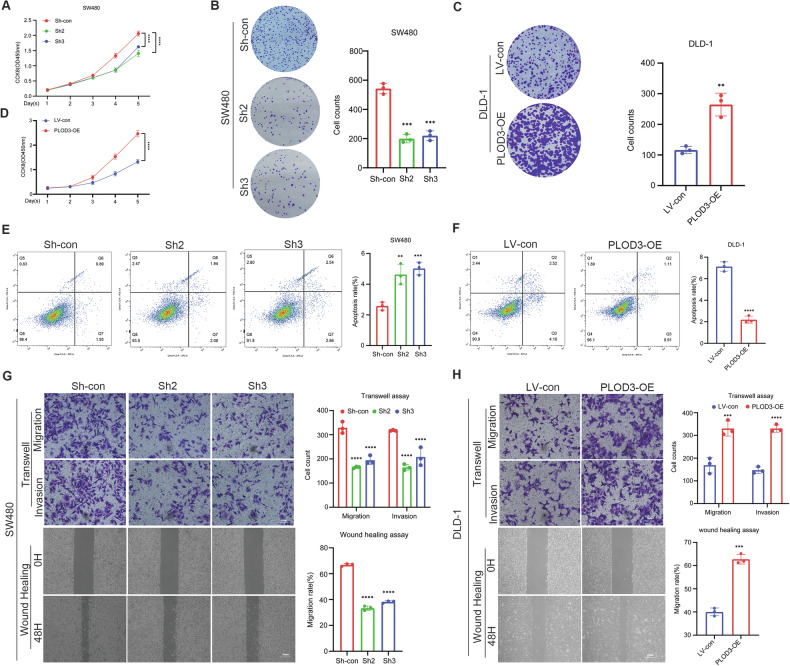
PLOD3 promotes CRC cell proliferation, migration and invasion in vitro. **A**, **D** CCK-8 assays were performed to evaluate the viability of PLOD3 knockdown or overexpression cells. **B**, **C** Colony formation assays were applied to detect the proliferation of CRC cells. **E**, **F** Flow cytometry was conducted to determine the apoptotic rates (LR + UR) of cells. **G**, **H** Transwell and wound healing assays were used to assess the abilities migration and invasion of CRC cells. LR, early apoptotic cells; UR, terminal apoptotic cells. Data are shown as the mean ± SD of three independent experiments, *P < 0.05, **P < 0.01, ***P < 0.001, ****P < 0.0001and *P* > 0.05, not significant (n.s.).

First, three shRNAs against PLOD3 were transfected into SW480 cells and a PLOD3 overexpression lentivirus was transfected into DLD-1 cells to induce stable expression of PLOD3, the transfection efficiency was verified via qRT‒PCR and western blotting (Fig. S1G, H). A variety of cell function assays were performed to elucidate the function of PLOD3 in the progression of CRC. CCK8 and colony formation assays confirmed that PLOD3 knockdown strongly suppressed SW480 cell proliferation (Fig. 3A, B), while PLOD3 overexpression strongly promoted DLD-1 cell proliferation (Fig. 3C, D). Apoptosis assays demonstrated that PLOD3 knocked down markedly increased the proportion of apoptotic cells (Fig. 3E). Conversely, the opposite phenomenon was observed when PLOD3 was overexpressed (Fig. 3F). Additionally, wound healing and transwell assays revealed that PLOD3 knockdown impaired SW480 cell migration and invasion (Fig. 3G); however, PLOD3 overexpression promoted DLD-1 cell migration and invasion (Fig. 3H). Collectively, these results indicated that overexpression of PLOD3 could enhance the proliferation, migration, and invasion ability of CRC cells in vitro.

### PLOD3 facilitates CRC cell proliferation and metastasis in vivo

**Fig. 4:**
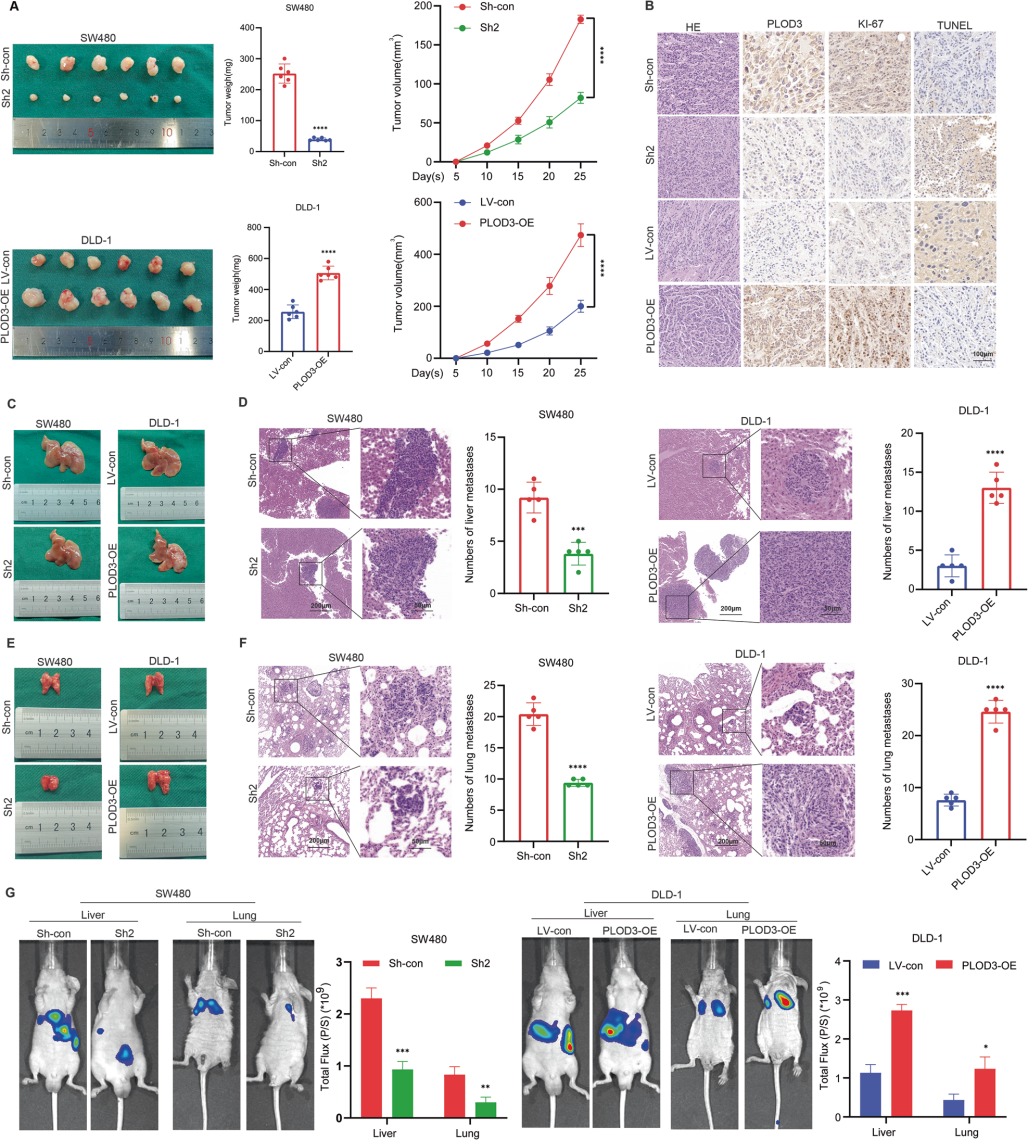
Effects of PLOD3 on CRC cells proliferation, migration, and invasion in vivo. **A** Images of xenograft tumors induced by the subcutaneous inoculation of nude mice. Tumor size and average weight were observed. **B** Representative H&E staining,PLOD3, Ki-67, and TUNEL immuno -staining of xenograft tumors. **C**, **E** Representative photographs of liver and lung metastases were obtained from nude mice. **D**, **F** H&E staining of liver and lung metastatic tumors. **G** Representative images and analysis of bioluminescent intensity in liver and lung metastases. All data are presented as the mean ± SD of three independent experiments, *P < 0.05, **P < 0.01, ***P  < 0.001, ****P < 0.0001 and *P* > 0.05, not significant (n.s.).

To further verify the effect of PLOD3 on CRC proliferation, a xenograft tumor model was generated using the stably transfected cells mentioned above. PLOD3 overexpression significantly increased tumor growth, as reflected by an obvious increase in tumor weight and volume compared with those in the control group. However, the opposite effect was observed with PLOD3 knockdown, with lower tumor volume and weight (Fig. 4A). Moreover, Ki67, PLOD3 and TUNEL were detected by IHC analysis. PLOD3 knockdown suppressed tumor proliferation and enhanced tumor apoptosis; in contrast, upregulation of PLOD3 facilitated tumor proliferation and dampened tumor apoptosis (Fig. 4B). Subsequently, we constructed liver and lung metastasis models to explore the role of PLOD3 in the process of CRC metastasis. A greater number of liver metastatic nodules and greater luciferase activity were detected in the PLOD3-overexpressing group, while fewer liver metastatic nodules and decreased luciferase activity were detected in the PLOD3-knockdown group and in the lungs (Fig. 4C–G). These findings suggest that PLOD3 enhances CRC cell proliferation and metastasis in vivo.

### PLOD3 knockdown attenuated colitis-associated tumorigenesis

**Fig. 5:**
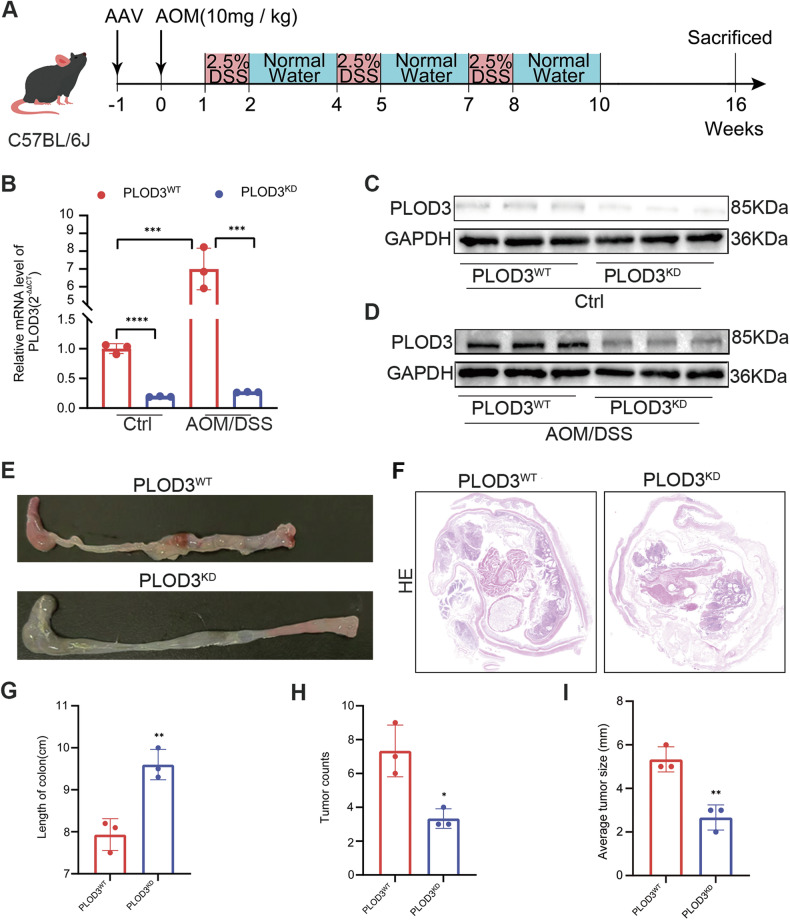
PLOD3 promotes colorectal tumorigenesis in vivo. **A** Scheme for the AOM/DSS-induced colon cancer model in C57BL/6J mice. **B**–**D** The mRNA and protein levels of PLOD3 in colon from the mice treated with or without AOM/DSS were measured by qRT‒PCR and western blot. **E** Representative photograph of colon tissues in PLOD3^WT^ and PLOD3^KD^ mice. **F** Hematoxylin and eosin (H&E) staining of colon tumors from PLOD3^WT^ And PLOD3^KD^ mice. **G**–**I** The length of the colon, tumor number and average tumor size in the PLOD3^WT^ and PLOD3^KD^ groups were analyzed. All data are presented as the mean ± SD of three independent experiments, *P < 0.05, **P < 0.01, ***P < 0.001, ****P < 0.0001 and *P* > 0.05, not significant (n.s.).

Encouraged by the aforementioned results, we investigated the therapeutic potential of PLOD3 knockdown on the initiation of CRC by employing a mouse model of colitis-associated tumorigenesis induced by AOM/DSS. Specifically, we administered AAV9-sh-PLOD3 or AAV9-sh-control via tail vein injection one week prior to the establishment of the model (Fig. 5A). The results confirmed the satisfactory efficiency of PLOD3 knockdown, as confirmed by western blot and qRT‒PCR assays (Fig. 5B, C). Furthermore, our findings revealed that the expression of PLOD3 in the colon of mice treated with AOM/DSS was significantly higher in the colon of control mice (Fig. 5D). Notably, knockdown of PLOD3 markedly hindered the development of CRC tumors, as evidenced by the reduced number and size of tumors in the PLOD3 knockdown group compared to those in the control group, as determined by gross examination (Fig. 5E). Moreover, histological examination via H&E staining was performed to validate the results (Fig. 5F). Additionally, the length of the colon in the control group was reduced, however, the number and the size of the tumor in the PLOD3 knockdown group is significant decreased (Fig. 5G–I). In conclusion, our study suggested that targeting PLOD3 may be a potential therapeutic strategy for preventing CRC initiation in a colitis-associated setting.

### PLOD3 enhances CRC cell proliferation, invasion and migration by promoting autophagy

**Fig. 6:**
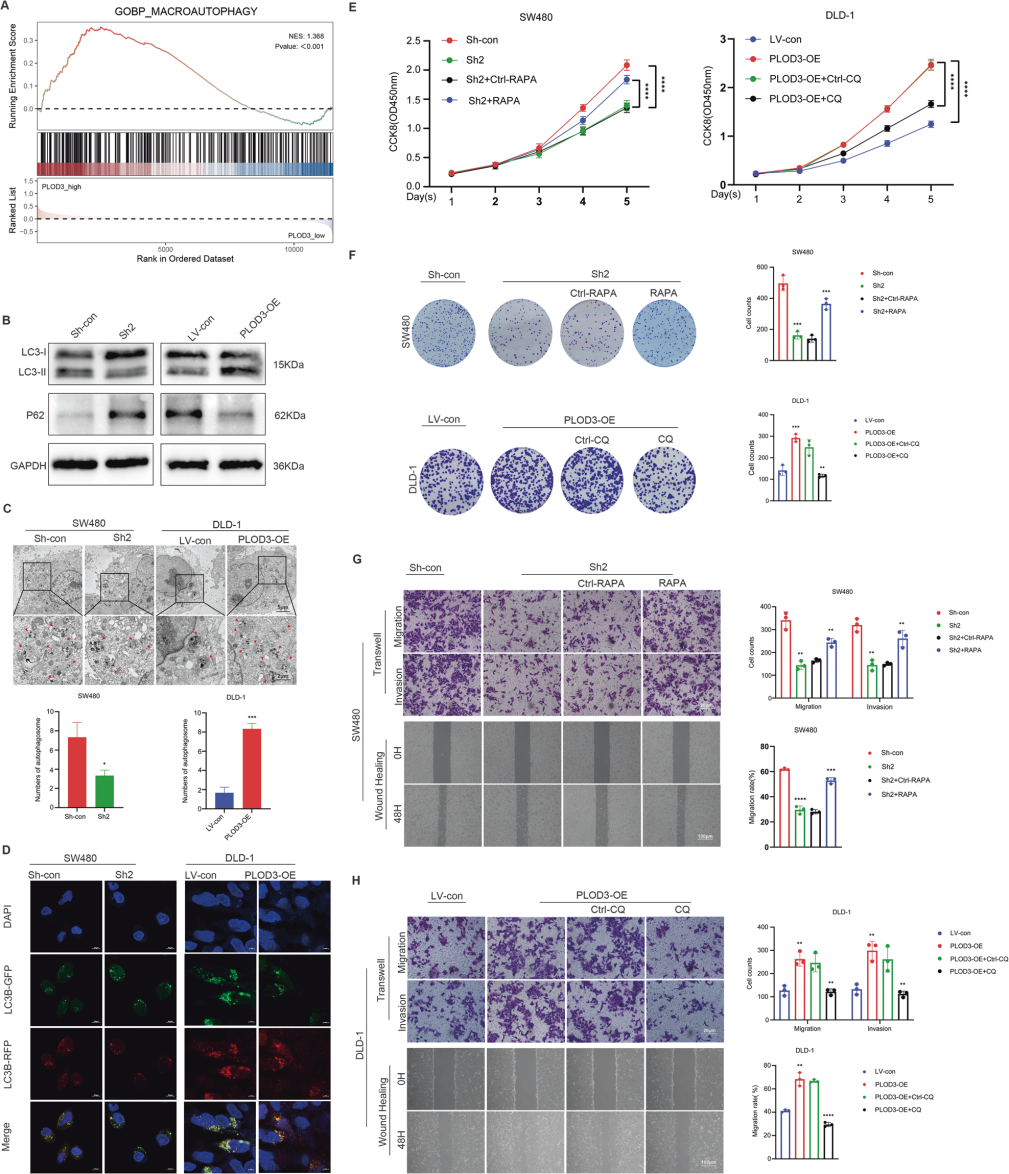
PLOD3 enhanced CRC progression by promoting autophagy. **A** Macroautophagy pathway was enriched in the dataset of GSE17356 by GSEA analysis. **B** WB was used to determine the protein expression levels of LC3B and P62 after PLOD3 was knocked down or overexpressed in CRC cells. **C** Representative image of autophagosomes after PLOD3 knockdown or over- expression in CRC cells. **D** Autophagic flux was assessed after PLOD3 overexpression or knocked down. **E**, **F** CCK8 and colony formation assays were conducted to detect the proliferation abilities of stably transfected CRC cells treated with CQ (10 µM) or RAPA (100 nM) for 24 h. **G**, **H** Transwell and wound healing assays were performed to evaluate the migration and invasion of stably transfected CRC cells treated with CQ (10 µM) or RAPA (100 nM) for 24 h.All data are shown as the mean ± SD of three independent experiments; *P  < 0.05, **P < 0.01, ***P < 0.001, ****P < 0.0001 and *P* > 0.05, not significant (n.s.).

To investigate the underlying mechanism, GSEA was performed to identify pathways associated with PLOD3-related genes. PLOD3-related genes were determined using Pearson correlation analysis, selecting the top 1000 genes most strongly correlated with PLOD3 in the TCGA-COAD dataset. The bar plot demonstrated that PLOD3-related genes were enriched in several pathways, including mitophagy, lysosome, mTOR signaling pathway, and autophagy (Fig. S1M). Notably, both mitophagy and autophagy belongs to macroautophagy. Furthermore, Previous studies have indicated that the biological function of PLOD3 may rely on the autophagy pathway [17]. To validate this, we analyzed the enriched Gene Ontology Biological Process (GO BP) pathways between the PLOD3 high-expression and low-expression groups in the GSE17356 and GSE29621 datasets. The macroautophagy pathway was significantly enriched in the PLOD3 high-expression group, with NES values of (1.368, 1.396) and *p*-values of (<0.001, 0.02) (Figs. 6A, S1N), respectively. These findings indicated that PLOD3 was associated with autophagy and may influence the progression of colorectal cancer through the autophagy pathway. Subsequently, western blot was used to assess the protein level of P62 and the LC3BII/I ratio. As anticipated, P62 levels decreased, while the LC3BII/I ratio increased following PLOD3 overexpression in DLD-1 cells. Conversely, the opposite results were observed in SW480 cells after PLOD3 knockdown (Fig. 6B). Additionally, transmission electron microscopy (TEM) was employed to further explore the findings, as the results indicated that more autophagosomes were observed in DLD-1 cells after PLOD3 overexpression; however, the number of autophagosomes was reduced when PLOD3 was downregulated in SW480 cells (Fig. 6C). In addition, we used the RFP-GFP-LC3B reporter system to assess changes in autophagic flux. PLOD3 overexpression significantly promoted autophagic flux, as indicated by more red dots than yellow dots; however, autophagic flux was dampened after PLOD3 knockdown (Fig. 6D). To validate whether PLOD3 enhances CRC progression by promoting autophagy, a series of phenotypic assays were performed. PLOD3 overexpression promoted DLD-1 cell proliferation, migration and invasion were abolished after the cells incubated with chloroquine (an autophagy inhibitor) for 24 h, and PLOD3 knockdown suppressed SW480 cell proliferation, migration and invasion, which was reversed by treatment with rapamycin (an autophagy stimulator) for 24 h (Fig. 6E–H).

Taken together, these results suggest that PLOD3 enhances CRC progression by promoting autophagy.

### PLOD3 interacted with TM9SF4 and positively regulated its protein level

**Fig. 7:**
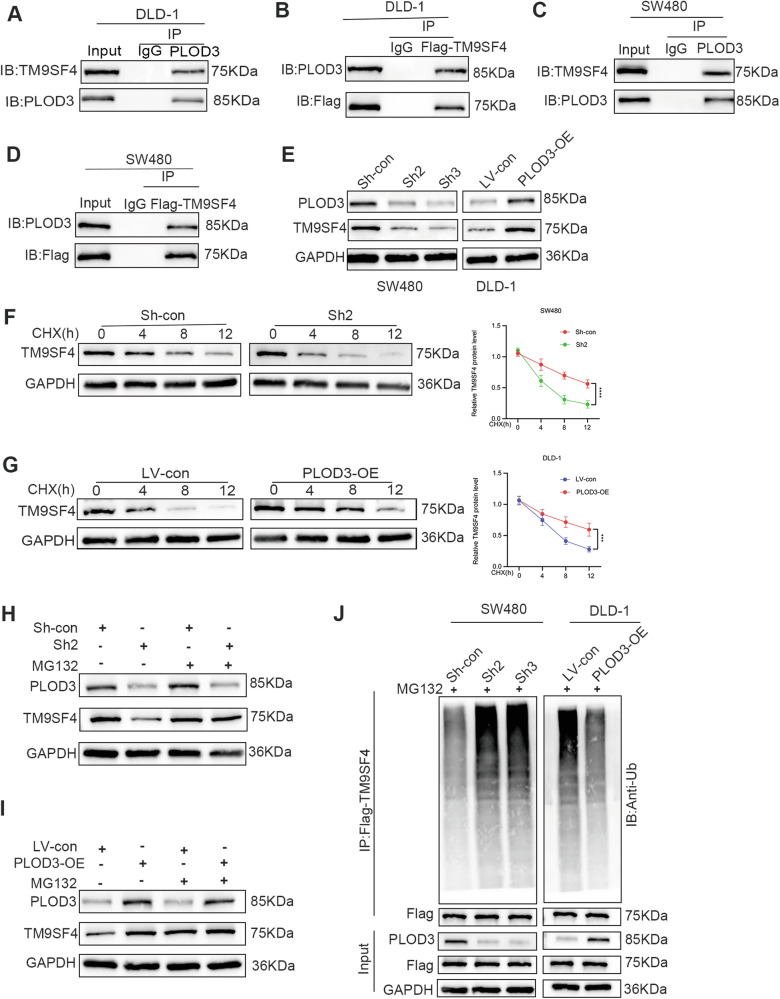
PLOD3 interacts with TM9SF4 and stabilizes TM9SF4 by preventing the degradation of TM9SF4 from ubiquitination. **A**–**D** The physical binding of PLOD3 and TM9SF4 in CRC cells transfected with FLAG-TM9SF4 were demonstrated by immunoprecipitation and WB analyses. **E** TM9SF4 protein levels in CRC cells with PLOD3 knockdown or upregulation. **F**, **G** TM9SF4 expression levels in CRC cells incubated with cycloheximide (CHX) for different durations and relative TM9SF4 protein levels. **H**, **I** TM9SF4 protein levels in PLOD3-knockdown or PLOD3-overexpressing CRC cells incubated with or without MG132. **J** Ubiquitination of TM9SF4 after PLOD3 was knocked down or overexpressed in CRC cells. All data are shown as the mean ± SD of three independent experiments, and *P* < 0.05 was considered to indicate statistical significance.

Secreted proteins normally exert their function by combining with membrane proteins. TM9SF4 is a transmembrane protein reported to be a novel molecule that promotes autophagy [30]; therefore, we hypothesized that PLOD3 promotes autophagy by interacting with TM9SF4. Then, co-IP assays were performed to examine the physical binding between PLOD3 and TM9SF4. The results showed that PLOD3 could interact with TM9SF4 (Fig. 7A–D). Next, we investigated whether PLOD3 could affect the expression of TM9SF4. Interestingly, the mRNA level of TM9SF4 did not significantly differ after PLOD3 knockdown or overexpressed (Fig. S1K, L); however, the protein level of TM9SF4 decreased following PLOD3 knockdown, and vice versa (Fig. 7E). CHX pulse-chase assays were conducted to determine the stability of TM9SF4 after PLOD3 downregulation or overexpression, and as indicated by the results, the protein stability of TM9SF4 was markedly impaired after PLOD3 knockdown (Fig. 7F); the opposite results were obtained after PLOD3 was upregulated (Fig. 7G). In addition, in cells treated with MG132, a proteasome inhibitor, the decrease in TM9SF4 levels induced by PLOD3 knockdown could be reversed (Fig. 7H, I), suggesting that the protein level of TM9SF4 may be regulated by PLOD3 through the proteasome pathway. Then, we conducted ubiquitination experiments to assess the ubiquitination level of TM9SF4 after PLOD3 was knocked down and overexpressed. The results demonstrated that overexpression of PLOD3 decreased the ubiquitination level of TM9SF4, while the ubiquitination level of TM9SF4 dramatically increased after PLOD3 knocked down (Fig. 7J).

Taken together, these findings strongly suggest that TM9SF4 interacts with PLOD3 and that its protein stability is dependent on PLOD3-induced ubiquitination.

### PLOD3-mediated promotion of autophagy depends on binding to TM9SF4 and enhancing CRC progression

Autophagy, a natural cellular process occurring in the cytoplasm, was investigated in relation to the subcellular localization of TM9SF4 and PLOD3. Immunofluorescence (IF) analysis revealed the colocalization of TM9SF4 and PLOD3 in the cytoplasm (Fig. S2A), leading us to hypothesize that TM9SF4 plays a crucial role in PLOD3-mediated autophagy in CRC. To test this hypothesis, we overexpressed TM9SF4 following PLOD3 knockdown and monitored changes in autophagic flux. Interestingly, we found that autophagy, which was impeded by the downregulation of PLOD3, was restored by the overexpression of TM9SF4. Furthermore, the proliferation, migration, and invasion of CRC cells, which were suppressed due to PLOD3 downregulation, were also rescued by TM9SF4 overexpression (Figs. S1J, S2B–E). Conversely, PLOD3 overexpression enhanced autophagy and promoted CRC cell proliferation, migration and invasion, and these effects were abolished by TM9SF4 knockdown (Figs. S1I, S3A–D).

Overall, the above results demonstrated that TM9SF4 plays a critical role in the process of PLOD3-mediated autophagy and enhanced CRC progression.

### Secreted PLOD3 promotes CRC cell migration and invasion in vitro and in vivo

**Fig. 8:**
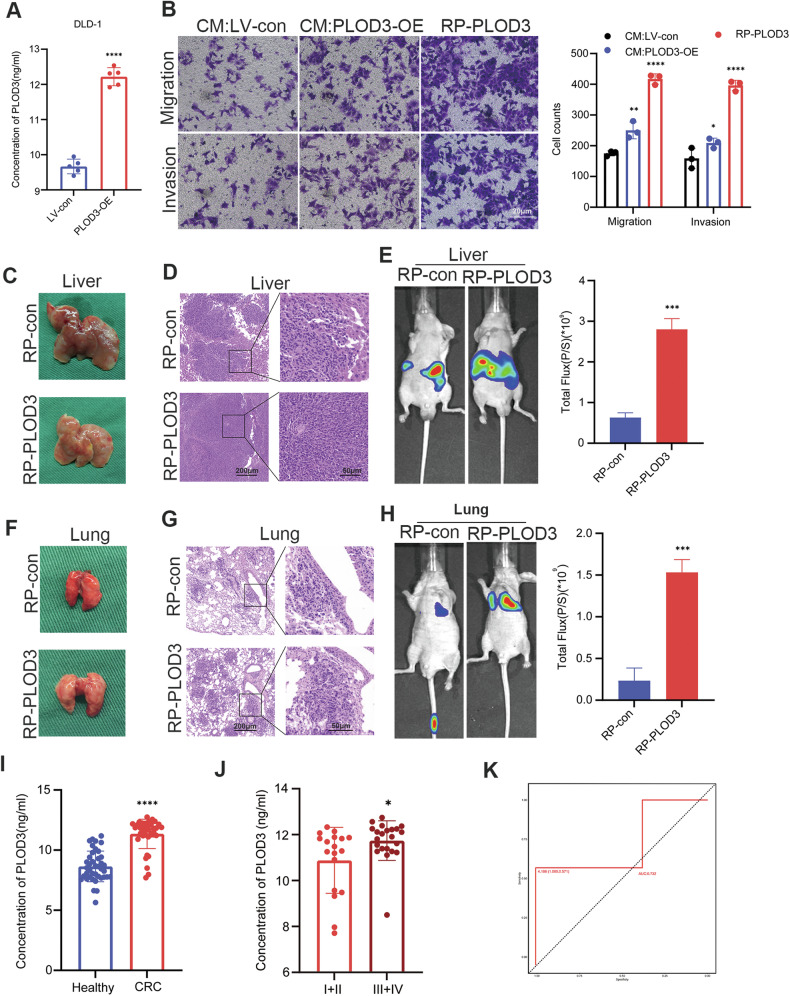
PLOD3 can be secreted by CRC cells, and secreted PLOD3 promotes CRC cell migration and invasion. **A** ELISA was conducted to determine the concentration of PLOD3 in the medium of CRC cells. **B** Transwell assays were performed to assess the migration and invasion abilities of CRC cells treated with different conditioned media and recombinant PLOD3. **C**, **F** Representative images of liver and lung metastases in mice injected with or without recombinant PLOD3. **D**, **G** H&E staining of liver and lung metastatic tumors.**E**, **H** Representative bioluminescence images of mice injected with or without recombinant PLOD3. **I** ELISA was conducted to determine the concentration of PLOD3 in the serum of CRC patients and healthy individuals. **J.** The correlation between PLOD3 levels in serum and tumor stage. **K** ROC curves were plotted to evaluate the sensitivity of PLOD3 as a biomarker for CRC diagnosis. CM conditional medium, RP recombinant protein. All data are shown as the mean ± SD of three independent experiments; *P < 0.05, **P < 0.01, ***P < 0.001, ****P < 0.0001 and *P* > 0.05, not significant (n.s.).

A previous study showed that PLOD3 can be secreted into the circulation in lung cancer [31]. Therefore, we investigated whether PLOD3 can be secreted and the function of secreted PLOD3 in the development of CRC. ELISA was performed to determine the concentration of PLOD3 in the medium of DLD-1 cells transfected with the PLOD3 overexpression plasmid and the vector. The results indicated that the concentration of PLOD3 in the medium of PLOD3-overexpressing DLD-1 cells was relatively higher than that in the medium of vector-transfected DLD-1 cells (Fig. 8A), suggesting that PLOD3 can be secreted by CRC cells.Then, conditional medium (CM) from PLOD3-overexpressing and vector-transfected DLD-1 cells was collected. Transwell assays demonstrated that CM from PLOD3-overexpressing DLD-1 cells promoted migration and invasion, as well as recombinant PLOD3 (Fig. 8B).

Next, we assessed the effect of the recombinant PLOD3 protein on metastasis in vivo. Recombinant PLOD3 protein or IgG was injected via the tail vein once every two days for two weeks before the liver metastasis mouse model was established. As the results indicated, more and larger metastatic nodules, as well as greater luciferase activity, were observed in the mice injected with the recombinant PLOD3 protein than in the control mice, and HE staining further confirmed these findings (Fig. 8C–H).

Additionally, we conducted a preliminary investigation to assess the potential of PLOD3 as a biomarker for CRC. Initially, we randomly selected 40 patients, including healthy individuals and patients diagnosed with CRC. ELISA experiments were then performed to measure the concentration of PLOD3 in the serum of both groups. The results indicated that the level of PLOD3 in the serum of CRC patients was significantly higher than that in the serum of healthy individuals (Fig. 8I), moreover, III + IV tumor stage group showed higher PLOD3 concentration in serum (Fig. 8J). To further evaluate the sensitivity of PLOD3 as a diagnostic biomarker for CRC, we generated a receiver operating characteristic (ROC) curve. The data revealed the promising diagnostic value of PLOD3 for CRC, with an AUC of 0.732 (Fig. 8K).

In summary, our findings suggest that PLOD3 can be secreted and that the secreted PLOD3 enhances CRC cell migration and invasion both in vitro and in vivo. Moreover, PLOD3 has emerged as a potential biomarker for CRC, demonstrating its significant diagnostic value.

## Discussion

Multiple studies have recently indicated that elevated levels of collagen in the ECM contribute to tumor progression. For instance, the knockout of collagen VI has been found to impede tumor formation and growth, while an increase in collagen I has been linked to the initiation and metastasis of breast cancer in various models [32–34]. Given that PLOD3 is the sole isozyme crucial for collagen synthesis and maturation, its procancer role has been established in several types of cancer [16, 17, 31]; however, the precise function and mechanism of PLOD3 in CRC remain incompletely understood. In our investigation, we discovered that hypomethylation of the PLOD3 promoter leads to the upregulation of PLOD3 in CRC tissues and cell lines. Notably, elevated PLOD3 expression is positively correlated with nervous system invasion, tumor stage, lymph node metastasis, and distant metastasis in CRC patients. Additionally, our findings revealed that PLOD3 enhances the proliferation, invasion, and metastasis of CRC cells both in vitro and in vivo. These results suggest that PLOD3 functions as an oncogene and may serve as a potential biomarker for predicting CRC prognosis.

Autophagy plays an important role in cancer progression [35]; however, the exact function of autophagy in CRC is still undefined. In our study, we demonstrated that PLOD3 promotes autophagy during the development of CRC; that overexpression of PLOD3 facilitates CRC cell proliferation, migration and invasion, which are dampened by treatment with chloroquine; and that rapamycin abolishes the repression of CRC proliferation, migration and invasion after PLOD3 knockdown. However, more attention has been given to the role of autophagy as a tumor suppressor, mainly because its suppression may promote the deregulated growth of tumor cells and contribute to tumor formation [36]. However, autophagy participates in the regulation of cancer metabolism by providing nutrients to cancer cells, especially in microenvironments with limited nutrients. This, in turn, promotes cancer progression once the tumor is initiated [37]. These findings are consistent with our results, suggesting that inhibiting autophagy may be a potential target for cancer treatment. In summary, the dual roles played by autophagy during cancer progression are not contradictory; the dominant role depends on the context involved [38]. Furthermore, during the process of autophagy during CRC progression, TM9SF4 was identified as a protein that interacts with PLOD3. TM9SF4 belongs to the TM9SF family and plays numerous roles in the development of various cancers [39–41]. Previous studies have also indicated that TM9SF4 promotes autophagy [30]. In agreement with these findings, our study demonstrated that TM9SF4 is crucial for PLOD3-mediated autophagy in CRC. Specifically, the overexpression of PLOD3, which enhances autophagy in CRC, is suppressed by the knockdown of TM9SF4. Conversely, the downregulation of PLOD3, which dampens autophagy, can be reversed by the expression of TM9SF4. Moreover, we found that PLOD3 stabilized the protein level of TM9SF4 by inhibiting polyubiquitination. Ubiquitination is an important post-translational modification and has been shown to participate in the regulation of various cancers [42, 43]. PLOD3 has been reported to inhibit FoxO3 through the ubiquitination degradation pathway [17], and along with our results, we speculated that PLOD3 may be a potential regulator of ubiquitination of the target protein.

Early detection is crucial to the prognosis of CRC patients. At present, colonoscopy remains the gold standard for CRC screening [44]; however, it is difficult for patients to accept this method because it is invasive. In contrast, the fecal occult blood test is more acceptable to patients, but the results are prone to false negatives or false positives because of various interfering factors for detecting colon polyps [45]. Therefore, identifying novel noninvasive biomarkers for CRC screening, such as CEA and CA199, is vital. In this study, we first reported that PLOD3 can be secreted by CRC cells and that secreted PLOD3 enhances CRC cell migration and invasion both in vitro and in vivo. More intriguingly, the serum level of PLOD3 was significantly elevated in patients diagnosed with CRC, indicating that this biomarker is more sensitive for detecting CRC than other serum biomarkers. Given its diagnostic and prognostic value, PLOD3 is expected to be a promising biomarker for CRC.

There are several limitations in our study. First, the underlying mechanism by which PLOD3-mediated ubiquitination stabilizes the protein TM9SF4 is not well understood. Second, although we showed that PLOD3 can be secreted by CRC cells and that PLOD3 promotes CRC cell migration and invasion both in vitro and in vivo, the mechanisms by which PLOD3 is secreted extracellularly to promote tumor progression need to be further investigated.

In summary, to our knowledge, few studies have focused on the relationship between PLOD3 and TM9SF4 in tumorigenesis. Our study demonstrated that PLOD3 acts as a key mediator of CRC progression by stabilizing the protein level of TM9SF4, thereby promoting autophagy. The PLOD3/TM9SF4 axis could be a promising therapeutic target for CRC.

## Availability of data and materials

The data in this study are available from the corresponding author upon reasonable request.

## Supplementary information

Figure S1

Figure S2

Figure S3

Table S1

Table S2

Supplementary figure and table legends

Original Data File

Original Data File
