# Correction: Hypomethylation induced overexpression of PLOD3 facilitates colorectal cancer progression through TM9SF4-mediated autophagy

**DOI:** 10.1038/s41419-025-07670-5

**Published:** 2025-05-06

**Authors:** Renzhong Zhu, Chuanxin Tian, Nan Gao, Zhiqiang Li, Sheng Yang, Yue Zhang, Ming Zhou, Kangpeng Jin, Chuan Zhang, Yueming Sun

**Affiliations:** 1 Institute of Translational Medicine, Medical College, Yangzhou University, Yangzhou, China; 2 Department of General Surgery, The First Affiliated Hospital of Nanjing Medical University, Nanjing, China; 3 The Colorectal Institute of Nanjing Medical University, Nanjing, China; 4 Jiangsu Province Engineering Research Center of Colorectal Cancer Precision Medicine and Translational Medicine, Nanjing, China; 5 General Surgery department of Dongtai People’s Hospital, Yancheng, China; 6 MOE Key Laboratory of Bioinformatics, Center for Synthetic and Systematic Biology, School of Life Sciences, Tsinghua University, Beijing, China

**Keywords:** Colorectal cancer

Correction to: *Cell Death and Disease* (2025)16:206 10.1038/s41419-025-07503-5, published online 25 March 2025

During the proof stage of the article, the author order was clearly defined with Professor Sun Yueming as the main corresponding author, placed at the end of all authors. This order was confirmed by our author team at that time and also aligns with our recognition of the contributions to the article. However, to our surprise, after the article was published online, Professor Sun Yueming's position has changed to the third from the last:

Renzhong Zhu^1,7^, Chuanxin Tian^2,3,4,7^, Nan Gao^5,7^, Zhiqiang Li^6,7^, Sheng Yang^2,3,4^, Yue Zhang^2,3,4^, Ming Zhou^1^, Yueming Sun^1,2,3,4,*^, Chuan Zhang^2,3,4,**^ and Kangpeng Jin^2,3,4^

Order of corresponding authors: jinkangpeng@njmu.edu.cn; zcsdly@126.com; sunyueming@njmu.edu.cn

Therefore, we request that the order of the authors be corrected to Renzhong Zhu^1,7^, Chuanxin Tian^2,3,4,7^, Nan Gao^5,7^, Zhiqiang Li^6,7^, Sheng Yang^2,3,4^, Yue Zhang^2,3,4^, Ming Zhou^1^, and Kangpeng Jin^2,3,4^, Chuan Zhang^2,3,4^, Yueming Sun^1,2,3,4^.

The order of the corresponding email: sunyueming@njmu.edu.cn; zcsdly@126.com; jinkangpeng@njmu.edu.cn

The original article has been corrected.

