# Supplementary figures and images for: Hypomethylation induced overexpression of PLOD3 facilitates colorectal cancer progression through TM9SF4-mediated autophagy
Source: Cell Death Dis. 2025 Mar 25;16(1):206. doi: 10.1038/s41419-025-07503-5 (PMC11937244; doi:10.1038/s41419-025-07503-5)

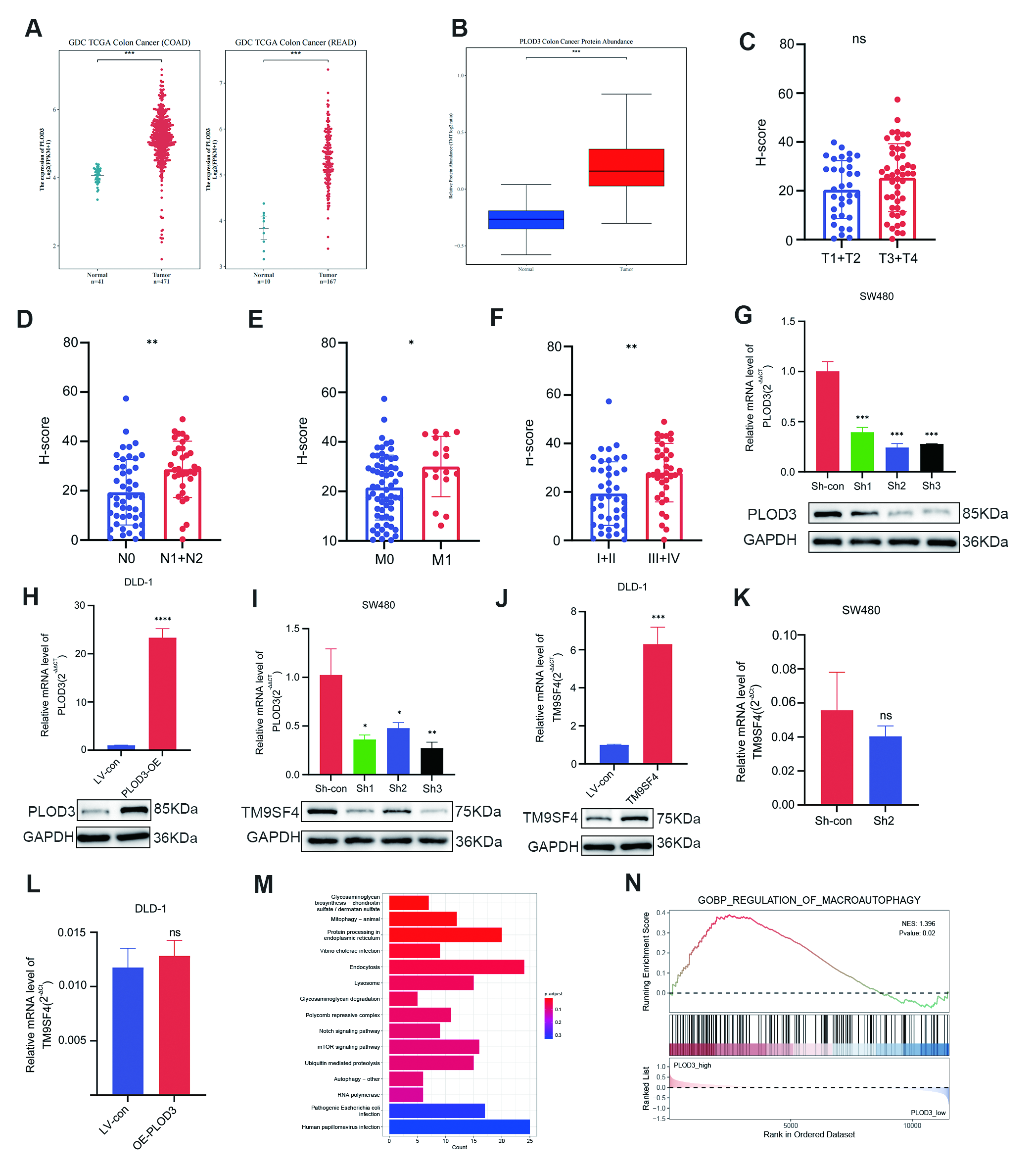

Supplement: Supplementary file 1 — Figure S1 [file 41419_2025_7503_MOESM1_ESM.tif]

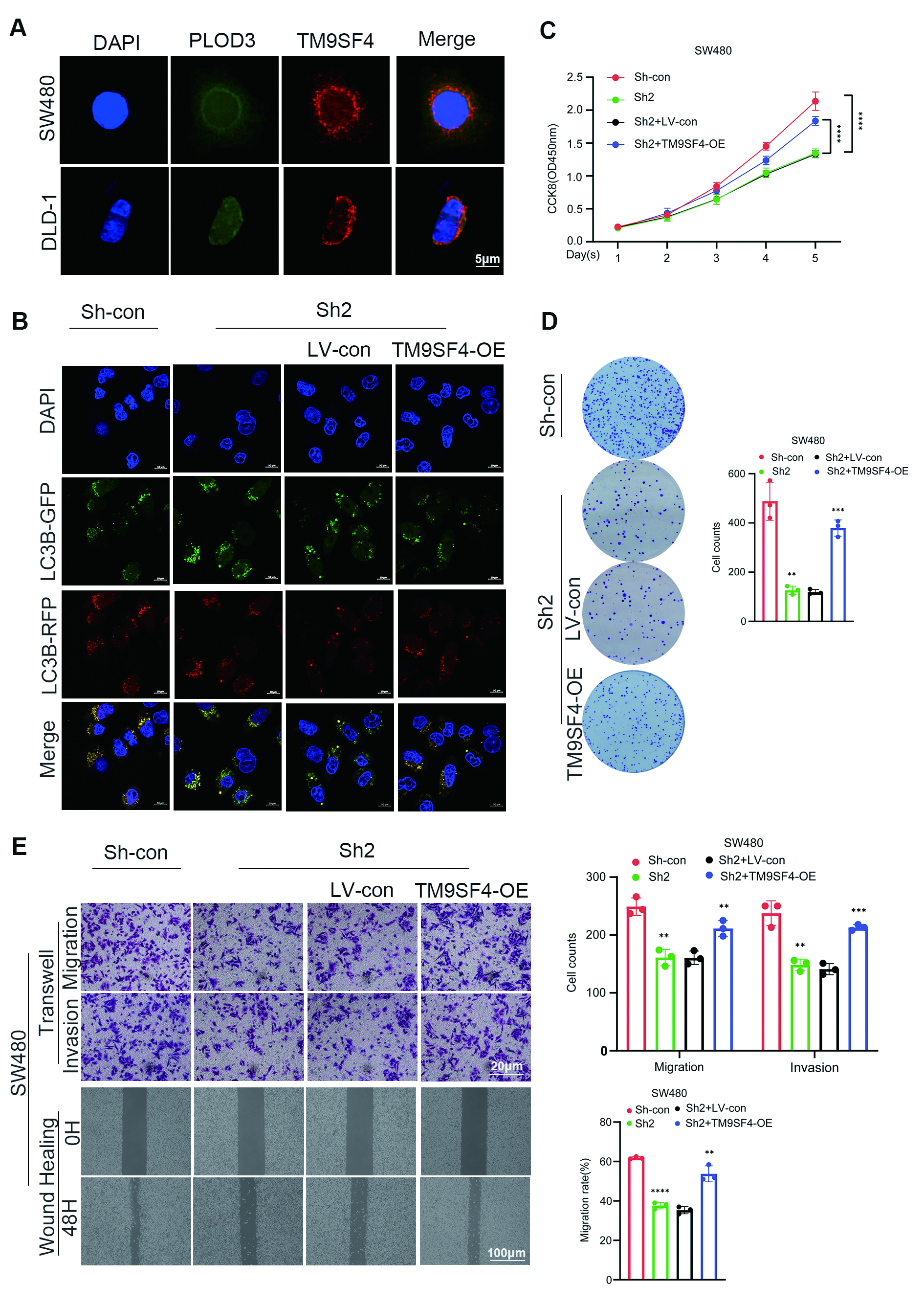

Supplement: Supplementary file 2 — Figure S2 [file 41419_2025_7503_MOESM2_ESM.tif]

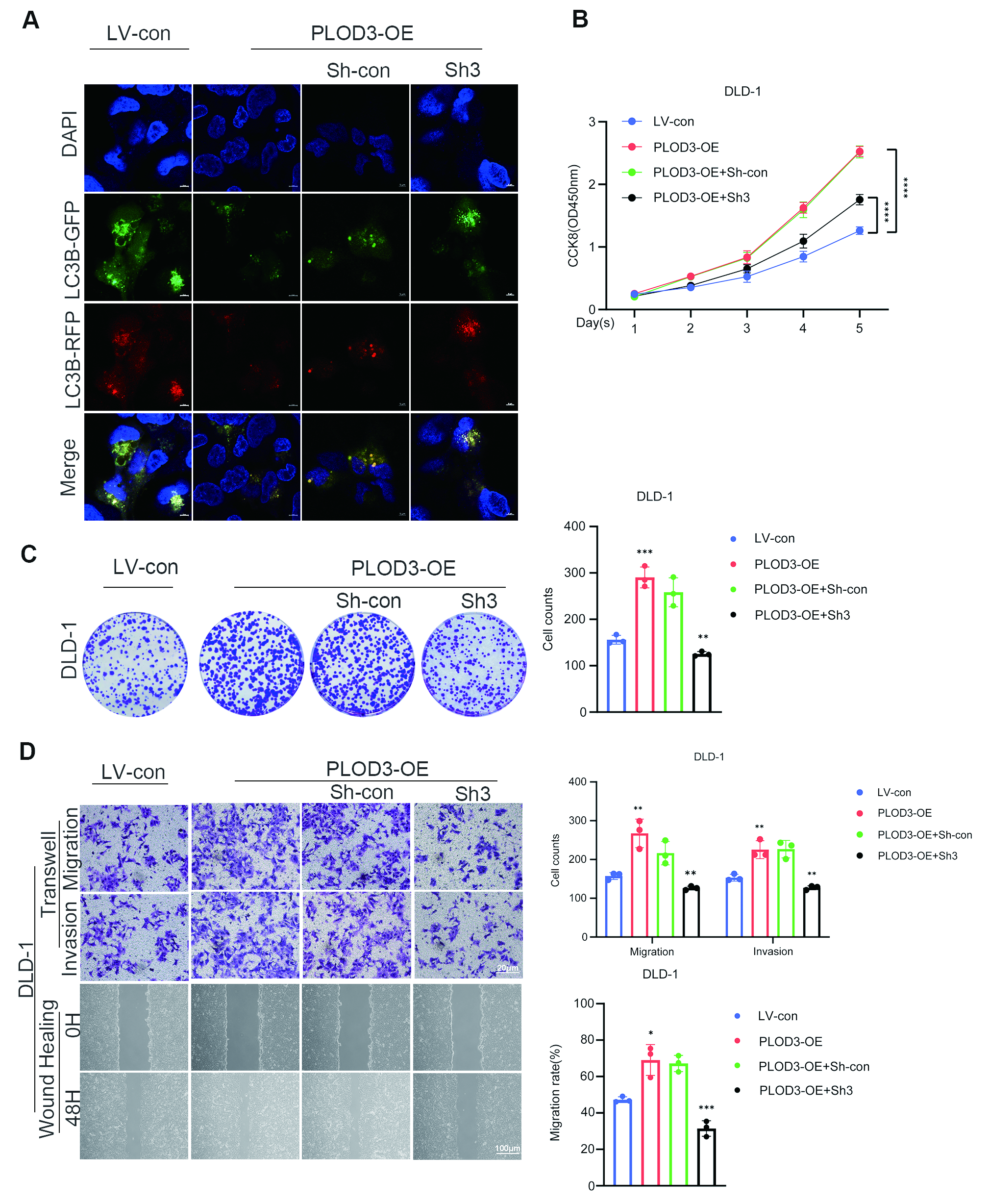

Supplement: Supplementary file 3 — Figure S3 [file 41419_2025_7503_MOESM3_ESM.tif]

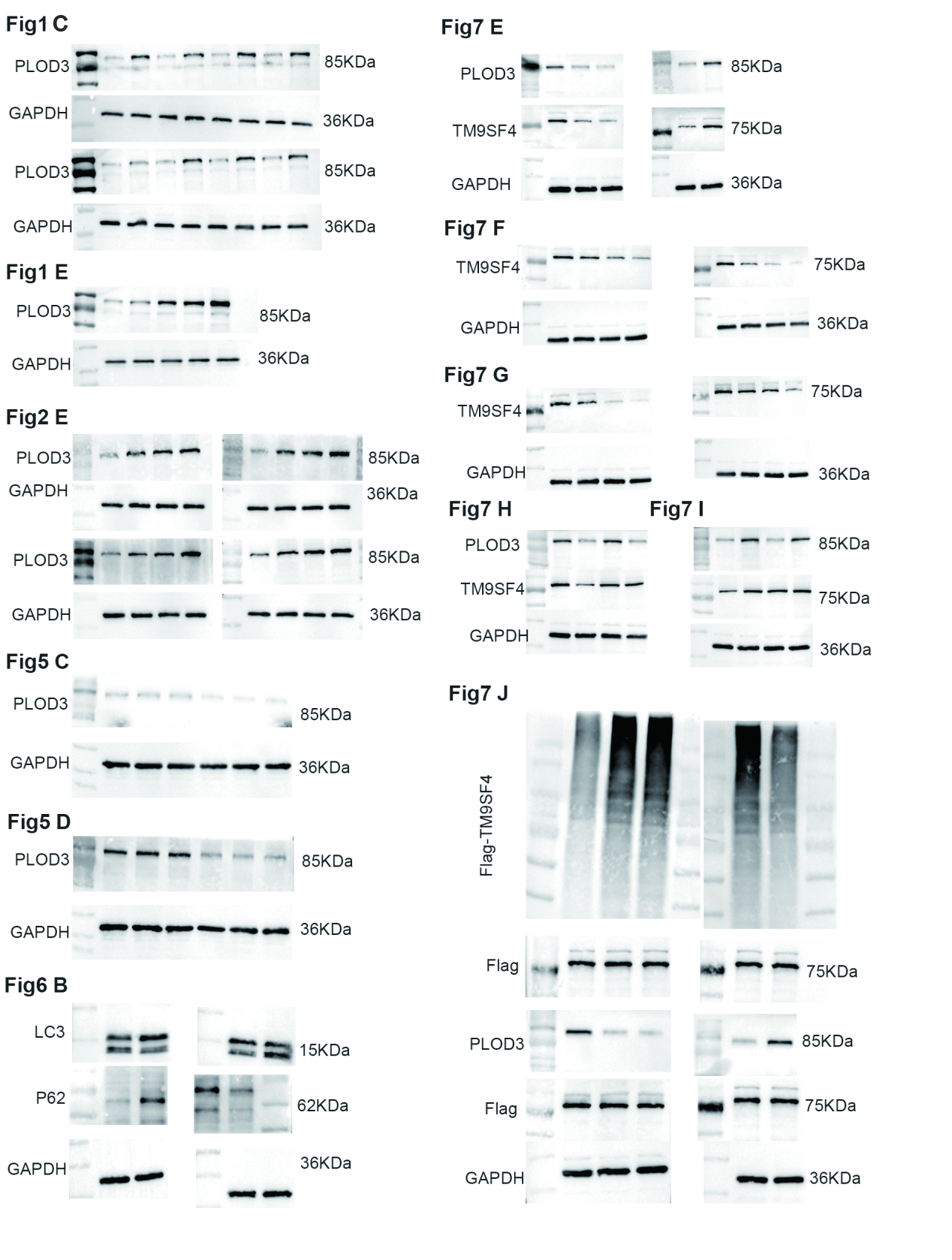

Supplement: Supplementary file 8 — Original Data File [file 41419_2025_7503_MOESM8_ESM.docx]
