# Supplementary material for: Hypomethylation induced overexpression of PLOD3 facilitates colorectal cancer progression through TM9SF4-mediated autophagy
Source: Cell Death Dis. 2025 Mar 25;16(1):206. doi: 10.1038/s41419-025-07503-5 (PMC11937244; doi:10.1038/s41419-025-07503-5)
Supplement: Supplementary file 4 — Table S1 [file 41419_2025_7503_MOESM4_ESM.docx]

**Table S1**

| qRT-PCR Primers |  |  |
| --- | --- | --- |
| Human-PLOD3 | Forward Primer | GACCCGGTCAACCCAGAGA |
|  | Reverse Primer | CTCCACCAACTGTTCGAGCC |
| Mouse-PLOD3 | Forward Primer | CCGTAACTTCTGCCTCCGAT |
|  | Reverse Primer | TGTTCGAGCCACATCACCC |
| TM9SF4 | Forward Primer | TCCGTTGTTGTGGTCTTC |
|  | Reverse Primer | GTCTTCAATGTCATCCTCCT |
| Human-GAPDH | Forward Primer | GGACCTGACCTGCCGTCTAG |
|  | Reverse Primer | GTAGCCCAGGATGCCCTTGA |
| Mouse-GAPDH | Forward Primer | GGAGAGTGTTTCCTCGTCCC |
|  | Reverse Primer | GATGGGCTTCCCGTTGATGA |
|  |  |  |
| short hairpin RNAs (shRNAs) sequence |  |  |
| Sh-PLOD3 #1 (87013GI-P) | Sense | CCGGGAGGATATGATCATCAT |
|  | Antisense | ATGATGATCATATCCTCCCGG |
| Sh-PLOD3 #2 (87014GG-P) | Sense | CTTCCTCAATTCTGGTGGATT |
|  | Antisense | AATCCACCAGAATTGAGGAAG |
| Sh-PLOD3 #3 (87016GL-P) | Sense | CTGAGCAATCAGCATGAATTT |
|  | Antisense | AAATTCATGCTGATTGCTCAG |
| Sh-TM9SF4 #1 (87017GM-P) | Sense | CCTCTCATTCATCCTTTACTA |
|  | Antisense | TAGTAAAGGATGAATGAGAGG |
| Sh-TM9SF4 #2 (87018GN-P) | Sense | GCGGATCACAGAAGACTACTA |
|  | Antisense | TAGTAGTCTTCTGTGATCCGC |
| Sh-TM9SF4 #3 (87019GO-P) | Sense | CTCGAACCCAGCTACCTTATG |
|  | Antisense | CATAAGGTAGCTGGGTTCGAG |
| Mouse-Sh-PLOD3 (80605GW-AAV) | Sense | GGTGTACCACGAGCCTCATAT |
|  | Antisense | ATATGAGGCTCGTGGTACACC |
|  |  |  |
| MSP primers |  |  |
| PLOD3 | Methylated Forward Primer | TTTTAGGTTTTCGTTTCGCGGC |
|  | Methylated Reverse Primer | TAAACCAACCGCTTAACACATATA |
|  | Unmethylated Forward Primer | TTTTTTAGGTTTTTGTTTTGTGGT |
|  | Unmethylated Reverse Primer | TAAACCAACCACTTAACACATATA |
|  |  |  |
| BSP primers |  |  |
| PLOD3 | Forward Primer | GGATTGTTAAAGAAAGTGGTTGGT |
|  | Reverse Primer | AACAAAAACTCTAAACTAAACCAACC |
