# Supplementary material for: Hypomethylation induced overexpression of PLOD3 facilitates colorectal cancer progression through TM9SF4-mediated autophagy
Source: Cell Death Dis. 2025 Mar 25;16(1):206. doi: 10.1038/s41419-025-07503-5 (PMC11937244; doi:10.1038/s41419-025-07503-5)
Supplement: Supplementary file 5 — Table S2 [file 41419_2025_7503_MOESM5_ESM.docx]

| **Table S2** | | |
| --- | --- | --- |
| **Antigens** | **Manufacturer** | **Application** |
| PLOD3 | Proteintech:11027-1-AP | 1:5000 for WB; 1:200 for IHC; 2μg for IP |
| TM9SF4 | Proteintech: ab140688 | 1:2000 for WB; 1:1000 for IHC;1:100 for IF |
| LC3 | CST:12741S | 1:2000 for WB; 1:100 for IF |
| P62 | CST: 88588S | 1:1000 for WB;1:400 for IF |
| Ki-67 | Abcam: ab15580 | 1:500 for IHC |
| GAPDH | Abcam: ab9485 | 1:10000 for WB |
| Flag | CST: #14793 | 1:1000 for WB; 1:50 for IP |
