# Supplementary material for: Hypomethylation induced overexpression of PLOD3 facilitates colorectal cancer progression through TM9SF4-mediated autophagy
Source: Cell Death Dis. 2025 Mar 25;16(1):206. doi: 10.1038/s41419-025-07503-5 (PMC11937244; doi:10.1038/s41419-025-07503-5)
Supplement: Supplementary file 6 — Supplementary figure and table legends [file 41419_2025_7503_MOESM6_ESM.docx]

**Supplementary figure and table legends**

Suppl. Figure 1. The expression of PLOD3 in public datasets. A. The mRNA level of PLOD3 based on TCGA and GEO datasets. B. The protein level of PLOD3 based on CPTAC datasets. C-F. Correlation of PLOD3 expression with clinical features based on the TMA. G, H. The transfection efficiency of PLOD3 in SW480 and DLD-1 cells was verified by qRT‒PCR and WB. I, J. qRT‒PCR and WB were conducted to detect the transfection efficiency of TM9SF4 in SW480 and DLD-1 cells. K,L. The relative mRNA level of TM9SF4 after PLOD3 down-regulation or overexpression. M. autophagy pathway was involved in the progression of CRC by bioinformation analysis. N. Macroautophagy pathway was enriched in the dataset of GSE29621 by GSEA analysis. All data are shown as the mean ± SD of three independent experiments; *P < 0.05, **P < 0.01, ***P < 0.001, ****P < 0.0001 and P > 0.05, not significant (n.s.).

Suppl. Figure 2. PLOD3 promote autophagy depends on its binding to TM9SF4. A. PLOD3 and TM9SF4 were colocalized in the cytoplasm. B. PLOD3 knocked down dampened autophagic flux was reversed by TM9SF4 overexpression. C-E. TM9SF4 overexpression reversed the decreases in cell proliferation, migration and invasion caused by PLOD3 knockdown. All data are shown as the mean ± SD of three independent experiments; *P < 0.05, **P < 0.01, ***P < 0.001, ****P < 0.0001 and P > 0.05, not significant (n.s.).

Suppl. Figure 3. PLOD3 promotes autophagy by interacting with TM9SF4. A. TM9SF4 silencing reversed the increased autophagic flux caused by PLOD3 overexpression. B-D. TM9SF4 knockdown reversed the increase in proliferation, migration and invasion caused by PLOD3 overexpression. All data are presented as the mean ± SD of three independent experiments; *P < 0.05, **P < 0.01, ***P < 0.001, ****P < 0.0001 and P > 0.05, not significant (n.s.).

Supplemental Table 1.The primers used in this study.

Supplemental Table 2. The antibodies used in this study.

Supplementary Table 3. Original western blots.

Supplemental Table 4. Original qRT-PCR data
